# Supplementary material for: Question-answering system extracts information on injection drug use from clinical notes
Source: Commun Med (Lond). 2024 Apr 3;4:61. doi: 10.1038/s43856-024-00470-6 (PMC10991373; doi:10.1038/s43856-024-00470-6)
Supplement: Supplementary file 1 — Supplementary Material [file 43856_2024_470_MOESM1_ESM.pdf]

Supplementary Information

Title: Question-Answering System Extracts Information on Injection Drug Use from Clinical Notes

Authors: Maria Mahbub[1], Ian Goethert[2], Ioana Danciu[3,4], Kathryn Knight[2], Sudarshan Srinivasan[1], Suzanne Tamang[5,6], Karine Rozenberg-Ben-Dror[7], Hugo Solares[5], Susana Martins[5], Jodie Trafton[5], Edmon Begoli[1], and Gregory D. Peterson[8]

Affiliations:

1. Cyber Resilience and Intelligence Division, Oak Ridge National Laboratory, Oak Ridge, TN, USA
2. Information Technology Services Division, Oak Ridge National Laboratory, Oak Ridge, TN, USA
3. Computational Sciences and Engineering Division, Oak Ridge National Laboratory, Oak Ridge, TN, USA
4. Department of Biomedical Informatics, Vanderbilt University, Nashville, TN, USA
5. Program Evaluation and Resource Center, Office of Mental Health and Suicide Prevention, Department of Veterans Affairs, USA
6. Department of Medicine, Stanford University School of Medicine, Stanford, CA, USA
7. Veterans Affairs Great Lakes Health Care System, Westchester, IL, USA
8. Department of Electrical Engineering and Computer Science, University of Tennessee, Knoxville, Knoxville, TN, USA

Supplementary Tables

Supplementary Table 1: A detailed list of co-occurring phrases before/after IDU keywords along with the parsing rules, targeted pattern type, and examples of answers before and after parsing. Abbreviations: IDU, Injection Drug Use; h/o, history of; ivdu, intravenous drug use; hx, history; pmh, past medical history; hpi, history of present illness; sud, substance use disorder; oud, opioid use disorder.

**Targeted Patterns**	**Co-occurring Phrases Before/After IDU Keywords**	**Parsing Rules**	**Example Answers Before and After Parsing**
Negations (nullifying the existence of IDU/skin popping/track marks/needle sharing)	Negation Phrases (NP): den(ying/ies/ied), no, never	**Sentence:** <text before NP> NP <text after NP containing keywords><br>**Rule:** NP <text after NP containing keywords>	**Before:** 65y/o m w cardiac procedures, or recent surgical procedures, admits to drinking alcohol daily for the past 10 years, denies any history of ivdu<br>**After:** denies any history of ivdu
Temporal information (active/ historical/last/frequency of use)	Temporal Phrases (TemP): past/remote/distant/prior/ previous/former/active/ current/recent/last/long history/hx (or h/o), daily, occasional, past, remote, distant, prior, previous, former, active, current, recent, last, long, intermittent, hpi, history, hx, h/o, pmh	**Sentence:** <text before TemP> TemP <text after TemP containing keywords><br>**Rule:** TemP <text after TemP containing keywords>	**Before:** pt smokes cannabis, has a h/o ivdu but none now, went to rehab 2070<br>**After:** h/o ivdu but none now, went to rehab 2070
Additional temporal information	Additional Temporal Phrases (ATP): year(s)/yr(s)/month(s)/ mnth(s)/day(s)/d/wk/wks/ mos ago	**Sentence:** <TemP> <text after TemP and before ATP containing keywords> ATP <text after ATP><br>**Rule:** <TemP> <text after TemP and before ATP containing keywords> ATP	**Before:** last ivdu was 10 days ago, snorts cocaine occasionally<br>**After:** last ivdu was 10 days ago
Opioid/substance use disorder specific to IDU	Phrases related to Substance use disorder (SP): substance/polysubstance use/abuse disorder, sud, psud, oud, polysubstance, opioid use disorder, opioid, opiate	**Sentence:** <text before SP> SP <text after SP containing keywords><br>**Rule:** SP <text after SP containing keywords>	**Before:** 200m w niddm, htn, bipolar disorder and oud (iv heroin) on methadone maintenance, recent heroin relapse<br>**After:** oud (iv heroin) on methadone maintenance, recent heroin relapse
Status of track marks	Phrases related to Track Mark status (TMP): arm(s)/abnormal/multiple/ many/several/healing/healed /old/diffuse/localized/ visible/red/iv/fresh/dark/ needle/notable	**Sentence:** <text before TMP> TMP <text after TMP containing "track marks"><br>**Rule:** TMP <text after TMP containing "track marks">	**Before:** comments: extremities: mid line in upper right arm, scars and old track marks noted on mid arm<br>**After:** old track marks noted on mid arm

Supplementary Table 2: Mappings between the query groups and the words in gold-standard answers most likely to provide inquired information and example answers for each mapping.

**Query Groups**	**Words in Gold-standard Answers Most Likely to Provide Inquired Information**	**Sample Answers**
Drug names	IV drug names from Table 2, opioid, opiate, oud	recent ivdu with meth and heroin
Visible signs of IDU	Phrases for visible signs of IDU from Table 2	multiple track marks over extremities
Risky needle-using behavior	Phrases for risky needle-using behavior from Table 2	h/o sharing needles with gf
Active/historical use	Temporal phrases from Supplementary Table 1, remission	active iv drug user up to day of admission
Frequency of use	daily, occasional, regularly, often, sometimes, frequently, intermittent	- iv cocaine daily, ~$5–40/day
Last use	Additional temporal phrases from Supplementary Table 1, last, quit, since, clean	last ivdu >30 years ago
Skin popping	Phrases for skin popping from Table 2	h/o drug injections - skin popping
Harm reduction interventions	Phrases for harm reduction interventions from Table 2	patient participates in clean syringe program
Existence of IDU	Negation phrases from Supplementary Table 1	denies any ivdu for many years
Existence of IDU	Remaining answers	iv drug user

Supplementary Table 3: Statistics of the gold-standard dataset.

**Property**	**Statistics**
#Patients	1145
#Notes	2323
#Notes per patient (average)	2.03
#Samples in dataset	17410
#QA per note (average)	7.49
Note length (in words)	1013.09 (average), 1029 (median), 1785 (max)
Question length (in words)	6.72 (average), 7 (median), 14 (max)
Answer length (in words)	7.52 (average), 6 (median), 64 (max)

Supplementary Table 4: Statistics of the additional test datasets built using Cohort-Short and Cohort-Long.

**Property**	**Statistics**	
	**Test Dataset (Cohort-Short)**	**Test Dataset (Cohort-Long)**
#Patients	100	100
#Notes	203	146
#Notes per patient (average)	2.03	1.46
#Samples in dataset	1985	1110
#QA per note (average)	9.78	7.60
Note length (in words)	1336.53 (average), 1117 (median), 5154 (max)	1618.86 (average), 1224 (median), 5747 (max)
Question length (in words)	6.51 (average), 7 (median), 14 (max)	6.92 (average), 7 (median), 14 (max)
Answer length (in words)	7.59 (average), 7 (median), 21 (max)	8.56 (average), 7 (median), 43 (max)

Supplementary Table 5: Examples of answers predicted by the QA model along with the question and the gold-standard answers.

**Question**	**Gold-standard Answer**	**Predicted Answer**
Has the pt ever injected drugs?	iv drug use: []yes [x]no	iv drug use: []yes [x]no
Which injection drugs?	iv heroin	attempted to overdose with the intent to die via iv heroin use
Does the patient have any physical evidence of IDU?	scars and old track marks	old track marks noted on mid arm
Does the pt have a h/o needle-sharing or using dirty needles?	sharing needles/injectin heroin/ cocain in the past	pos sharing needles
Is the patient actively using IV drugs?	h/o iv heroin use about 7 years ago one time, but denies any iv drug use since	remote h/o iv heroin use about 7 years ago one time, but denies any iv drug use since.
What is the frequency of pt's injection drug use?	daily heroin use with extensive ivdu	daily heroin use with extensive ivdu
Does the patient have any history of skin popping?	"skin popping" heroin 3-4 days ago	admits to "skin popping" heroin 3-4 days ago and showed the undersigned a keloid scar on his left hand due to shooting heroin
When has the pt last injected drugs?	quit iv heroin and cocaine x 6 weeks	quit iv heroin and cocaine x 6 weeks
Has the patient received any harm reduction interventions specific to IDU behaviors?	topic covered: hiv, viral hepatitis, safe sex practices, pre-exposure prophylaxis syringe service program, naloxone usage	syringe service program

Supplementary Figures

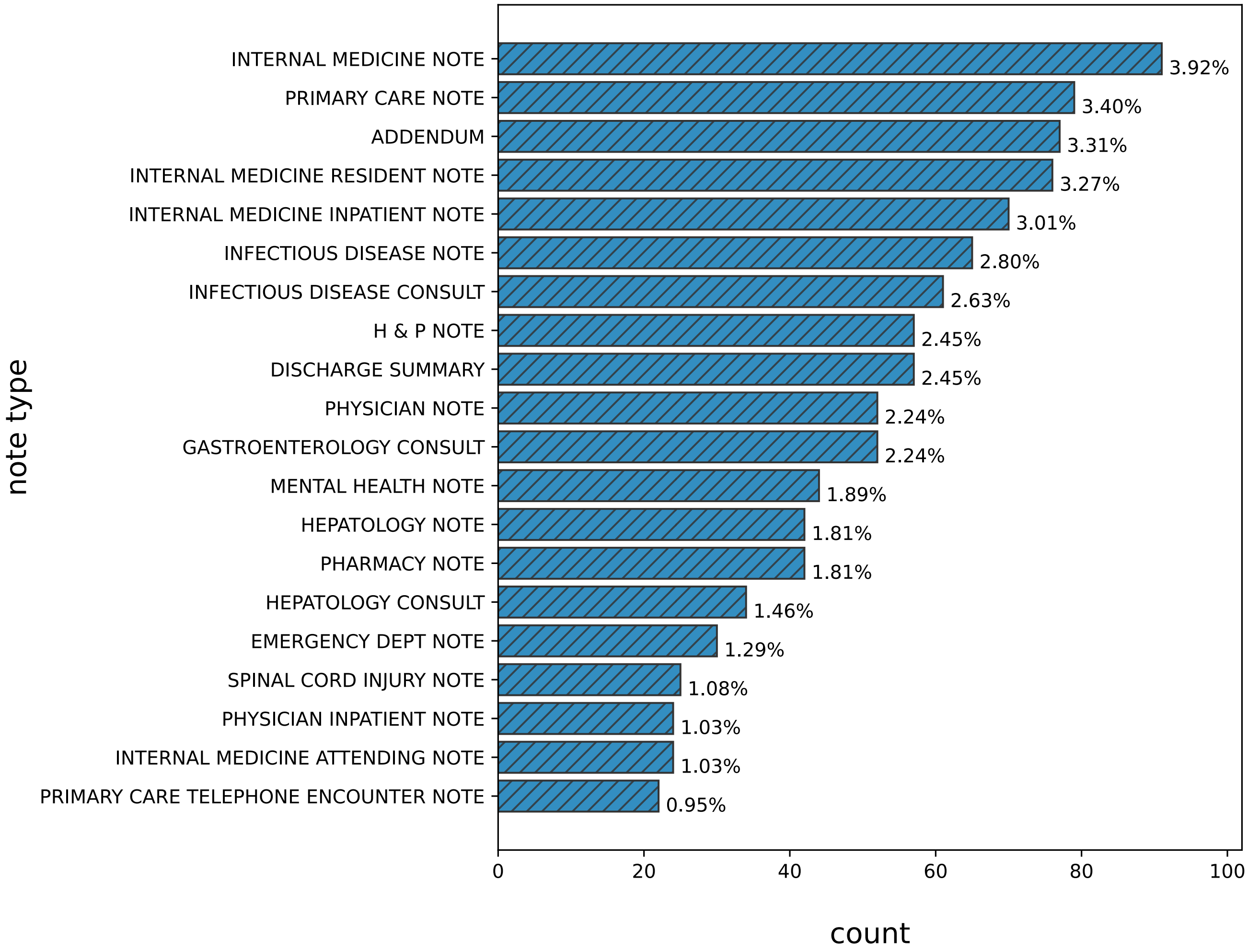

Supplementary Figure 1: Twenty most frequently encountered clinical note types in this study, along with their frequency distribution.

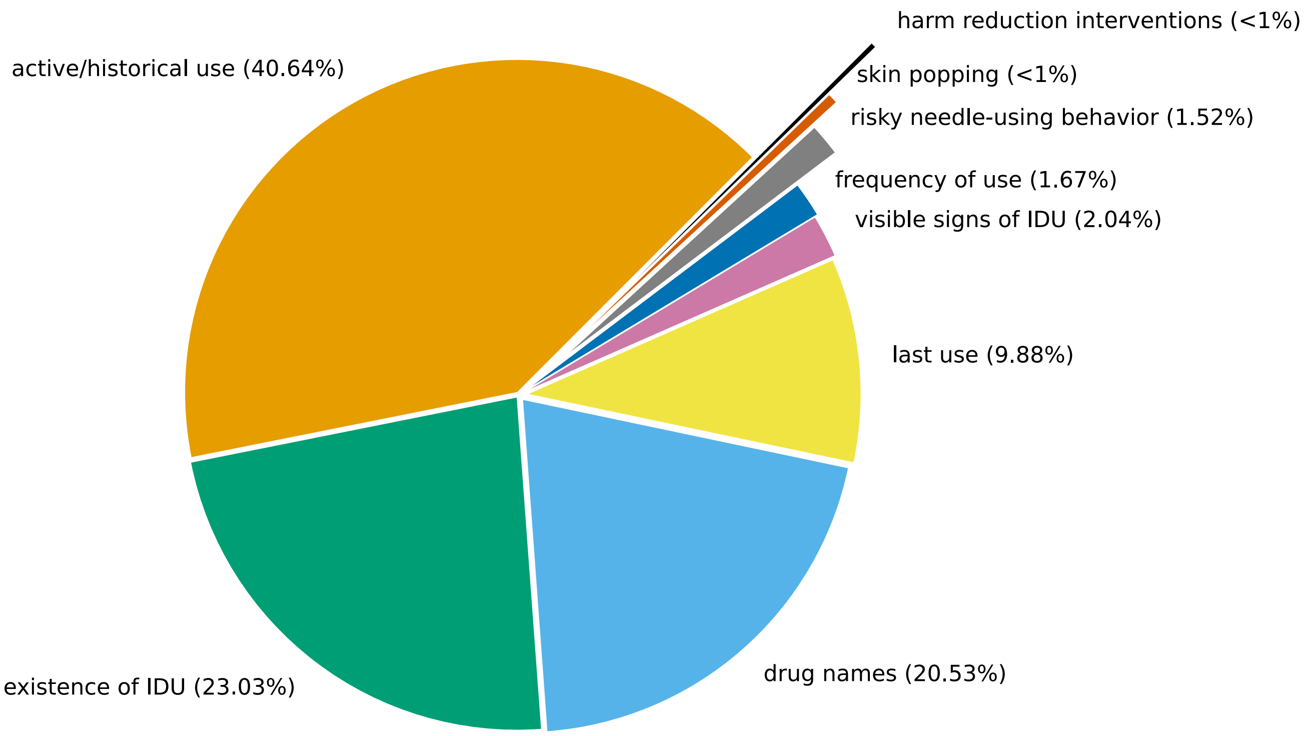

Supplementary Figure 2: Distribution of the query groups in the gold-standard dataset

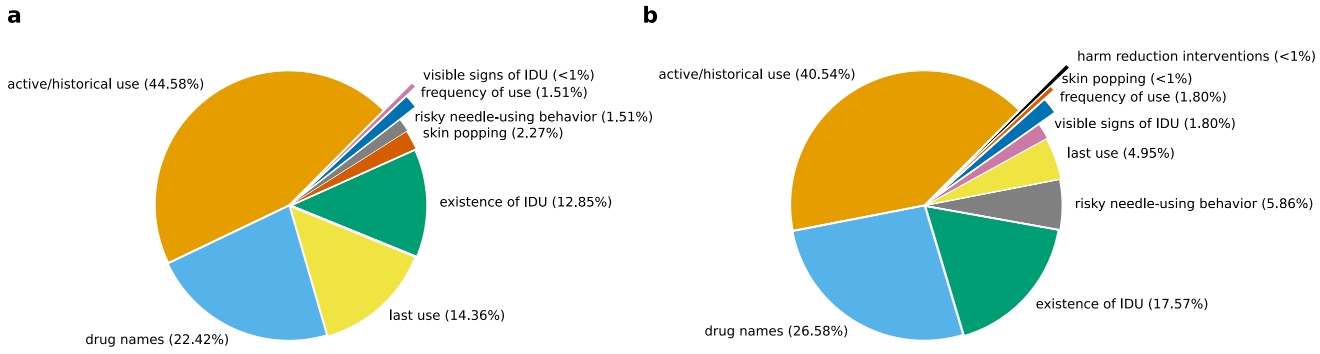

Supplementary Figure 3: Distribution of query groups in additional test datasets built using Cohort-Short (**a**) and Cohort-Long (**b**).
