# Supplementary material for: Question-answering system extracts information on injection drug use from clinical notes
Source: Commun Med (Lond). 2024 Apr 3;4:61. doi: 10.1038/s43856-024-00470-6 (PMC10991373; doi:10.1038/s43856-024-00470-6)
Supplement: Supplementary file 2 — Description of Additional Supplementary Files [file 43856_2024_470_MOESM2_ESM.pdf]

Description of Additional Supplementary Files

File Name: Supplementary Data 1
Description: Supplementary Data 1 contains the source data for Figure 3.

File Name: Supplementary Data 2
Description: Supplementary Data 2 contains the source data for Figure 4.
