# Supplementary material for: Question-answering system extracts information on injection drug use from clinical notes
Source: Commun Med (Lond). 2024 Apr 3;4:61. doi: 10.1038/s43856-024-00470-6 (PMC10991373; doi:10.1038/s43856-024-00470-6)
Supplement: Supplementary file 5 — Reporting Summary [file 43856_2024_470_MOESM5_ESM.pdf]

nature portfolio

Corresponding author(s): Maria Mahbub

Last updated by author(s): Feb 29, 2024

Reporting Summary

Nature Portfolio wishes to improve the reproducibility of the work that we publish. This form provides structure for consistency and transparency in reporting. For further information on Nature Portfolio policies, see our Editorial Policies and the Editorial Policy Checklist.

Statistics

For all statistical analyses, confirm that the following items are present in the figure legend, table legend, main text, or Methods section.

n/a	Confirmed	
☐	☒	The exact sample size (*n*) for each experimental group/condition, given as a discrete number and unit of measurement
☒	☐	A statement on whether measurements were taken from distinct samples or whether the same sample was measured repeatedly
☒	☐	The statistical test(s) used AND whether they are one- or two-sided<br>*Only common tests should be described solely by name; describe more complex techniques in the Methods section.*
☒	☐	A description of all covariates tested
☒	☐	A description of any assumptions or corrections, such as tests of normality and adjustment for multiple comparisons
☒	☐	A full description of the statistical parameters including central tendency (e.g. means) or other basic estimates (e.g. regression coefficient) AND variation (e.g. standard deviation) or associated estimates of uncertainty (e.g. confidence intervals)
☒	☐	For null hypothesis testing, the test statistic (e.g. *F*, *t*, *r*) with confidence intervals, effect sizes, degrees of freedom and *P* value noted<br>*Give P values as exact values whenever suitable.*
☒	☐	For Bayesian analysis, information on the choice of priors and Markov chain Monte Carlo settings
☒	☐	For hierarchical and complex designs, identification of the appropriate level for tests and full reporting of outcomes
☒	☐	Estimates of effect sizes (e.g. Cohen's *d*, Pearson's *r*), indicating how they were calculated

Our web collection on statistics for biologists contains articles on many of the points above.

Software and code

Policy information about availability of computer code

Data collection	The raw notes were curated from VA CDW.
Data analysis	The code used to develop the QA models is a modified version of the publicly available huggingface example for the question-answering task, which can be found here: https://github.com/huggingface/transformers/blob/master/examples/legacy/question-answering/run_squad.py.The modified code is stored in a GitHub repository at https://github.com/mmahbub/qa-system-for-injection-drug-use.

For manuscripts utilizing custom algorithms or software that are central to the research but not yet described in published literature, software must be made available to editors and reviewers. We strongly encourage code deposition in a community repository (e.g. GitHub). See the Nature Portfolio guidelines for submitting code & software for further information.

Data

Policy information about availability of data

All manuscripts must include a data availability statement. This statement should provide the following information, where applicable:

- Accession codes, unique identifiers, or web links for publicly available datasets
- A description of any restrictions on data availability
- For clinical datasets or third party data, please ensure that the statement adheres to our policy

The dataset developed for this study is not accessible to the public under requirements of the Health Insurance Portability and Accountability Act of 1996 and related privacy and security concerns. The underlying electronic health record data can only be used towards improving treatment for patients receiving services

from the Veterans Health Administration (VHA). Those interested in accessing VHA EHR data extracts curated for this quality improvement project to replicate and validate findings may contact the corresponding author regarding access via VHA collaboration. Source Data for Figures 3 and 4 is provided in Supplementary Data files 1 and 2, respectively.

Human research participants

Policy information about studies involving human research participants and Sex and Gender in Research.

Reporting on sex and gender	No reporting on sex and gender.
Population characteristics	No consideration of population characteristics.
Recruitment	No recruitment. This study focuses on developing a tool that can extract IDU-related information from any type of free-text clinical note.
Ethics oversight	This project was conducted as a national quality improvement effort to improve care for Veterans with substance use being treated in the Veterans Health Administration (VHA). Models were designed to be implemented into VHA decision support systems, and are not expected to be generalizable or valid for application outside of notes from the VHA Computerized Patient Record System (CPRS). As such, this work is considered non-research by VHA (as per ProgramGuide-1200-21-VHA-Operations-Activities.pdf (va.gov)). However, ORNL required additional oversight of this VHA clinical quality improvement project as local standard practice for all uses of patient medical record data within their institution, with approval of the project by the Oak Ridge National Laboratory IRB. The need for the veterans whose medical records were used in the study to give informed consent for the study was waived by the ORNL IRB.

Note that full information on the approval of the study protocol must also be provided in the manuscript.

Field-specific reporting

Please select the one below that is the best fit for your research. If you are not sure, read the appropriate sections before making your selection.

☐ Life sciences ☒ Behavioural & social sciences ☐ Ecological, evolutionary & environmental sciences

For a reference copy of the document with all sections, see nature.com/documents/nr-reporting-summary-flat.pdf

Behavioural & social sciences study design

All studies must disclose on these points even when the disclosure is negative.

Study description	Develop question-answering (QA) systems using natural language processing and deep learning to extract injection drug use (IDU)-related information from clinical notes.
Research sample	Sets of clinical notes, most likely containing IDU-related information, curated from VA CDW.
Sampling strategy	For additional testing of QA models, clinical notes from both February and November 2022 were utilized. A random sample of 200 patients and their respective notes were selected from the pool of patients with notes recorded during these months.
Data collection	No data collection was performed. The raw clinical notes were curated from VA CDW. The development of the QA datasets from the raw notes was performed using the methodology described in the paper.
Timing	Utilized clinical notes from January 2022 for the gold-standard dataset generation and model development. There is no specific reason for this selection. Clinical notes from February 2022 and November 2022 were selected for further testing of the QA models. The reason for selecting notes from February and November was to test the short-term and long-term information extraction capability of QA models.
Data exclusions	In the process of generating the gold-standard dataset for developing QA models capable of extracting IDU-related information from clinical notes, the study focused on excluding clinical notes that did not contain any information on IDU.
Non-participation	Not applicable.
Randomization	Random split for train, validation, and test sets from gold-standard QA dataset.

Reporting for specific materials, systems and methods

We require information from authors about some types of materials, experimental systems and methods used in many studies. Here, indicate whether each material, system or method listed is relevant to your study. If you are not sure if a list item applies to your research, read the appropriate section before selecting a response.

Materials & experimental systems

n/a	Involved in the study
☒	☐ Antibodies
☒	☐ Eukaryotic cell lines
☒	☐ Palaeontology and archaeology
☒	☐ Animals and other organisms
☐	☒ Clinical data
☒	☐ Dual use research of concern

Methods

n/a	Involved in the study
☒	☐ ChIP-seq
☒	☐ Flow cytometry
☒	☐ MRI-based neuroimaging

Clinical data

Policy information about clinical studies
All manuscripts should comply with the ICMJE guidelines for publication of clinical research and a completed CONSORT checklist must be included with all submissions.

Clinical trial registration	N/A. No clinical trial.
Study protocol	N/A. No study protocol.
Data collection	No data collection was performed. The raw notes were curated from VA CDW. The development of the QA datasets from the raw notes was performed using the methodology described in the paper.
Outcomes	The outcomes of this study are QA models capable of extracting IDU-related information from clinical notes and the pipeline to develop the QA models.
